# Health facility delivery among women of reproductive age in Nigeria: Does age at first birth matter?

**DOI:** 10.1371/journal.pone.0259250

**Published:** 2021-11-04

**Authors:** Obasanjo Afolabi Bolarinwa, Effiong Fortune, Richard Gyan Aboagye, Abdul-Aziz Seidu, Olalekan Seun Olagunju, Ugochinyere Ijeoma Nwagbara, Edward Kwabena Ameyaw, Bright Opoku Ahinkorah

**Affiliations:** 1 Department of Public Health Medicine, School of Nursing and Public Health, University of KwaZulu-Natal, Durban, South Africa; 2 Faculty of Social Sciences, Department of Demography and Social Statistics, Obafemi Awolowo University, Ile-Ife, Nigeria; 3 Department of Medical Laboratory Science, University of Calabar, Calabar, Nigeria; 4 School of Public Health, University of Health and Allied Sciences, Ho, Ghana; 5 College of Public Health, Medical and Veterinary Sciences, James Cook University, Townsville, Australia; 6 Department of Estate Management, Takoradi Technical University, Takoradi, Ghana; 7 School of Public Health University of Technology Sydney, Sydney, Australia; Queen’s University at Kingston, CANADA

## Abstract

**Background:**

High maternal mortality ratio in sub-Saharan Africa (SSA) has been linked to inadequate medical care for pregnant women due to limited health facility delivery utilization. Thus, this study, examined the association between age at first childbirth and health facility delivery among women of reproductive age in Nigeria.

**Methods:**

The study used the most recent secondary dataset from Nigeria’s Demographic and Health Survey (NDHS) conducted in 2018. Only women aged15-49 were considered for the study (N = 34,193). Bi-variate and multivariable logistic regression models were used to examine the association between age at first birth and place of delivery. The results were presented as crude odds ratios and adjusted odds ratios (aOR) with corresponding 95% confidence intervals (CIs). Statistical significance was set at p<0.05.

**Results:**

The results showed that the prevalence of health facility deliveries was 41% in Nigeria. Women who had their first birth below age 20 [aOR = 0.82; 95%(CI = 0.74–0.90)] were less likely to give birth at health facilities compared to those who had their first birth at age 20 and above.

**Conclusion:**

Our findings suggest the need to design interventions that will encourage women of reproductive age in Nigeria who are younger than 20 years to give birth in health facilities to avoid the risks of maternal complications associated with home delivery. Such interventions should include male involvement in antenatal care visits and the education of both partners and young women on the importance of health facility delivery.

## Background

The Sustainable Development Goal (SDG) 3 aims to ensure healthy lives and promote well-being for all ages [1]. However, high maternal mortality ratio (MMR), especially in low-and middle-income countries (LMICs), has remained worrisome [2–4]. In line with this, SDG 3.1 calls for the global MMR reduction to less than 70 per 100,000 live births by the year 2030 [1].

Although many countries have reduced their MMRs, causing a general global decline, efforts to reduce MMRs of countries in sub-Saharan Africa (SSA) have largely stagnated [5]. Nigeria’s maternal and child health indices are among the worst in the world. In 2015, the World Health Organization (WHO) estimated that 19% of global maternal deaths occurred in Nigeria [5], with an estimated MMR of 814 per 100,000 live births, and this placed a lifetime risk of maternal death at 1 in 22 in contrast to 1 in 4900 in developed countries [5]. However, the 2018 Nigeria Demographic Health Survey (NDHS) estimated MMR of 512 per 100,000 live births. Despite this reduction, Nigeria still remains one of the countries with the highest MMR globally [6].

Neonatal mortality is another serious public health threat in SSA [7]. In 2019, SSA had an estimate of 27 per 1000 live births, while Nigeria reported 36 per 1000 live births out of the estimate [8]. Similar to MMR, efforts to reduce neonatal mortality in SSA, especially in Nigeria, have been relatively slow [9]. Between 2010 and 2019, only a 13% reduction in neonatal mortality ratio was achieved in Nigeria [8,10]. The high prevalence of maternal and neonatal deaths in SSA and Nigeria has been linked to inadequate medical care for pregnant women, resulting in low health facility delivery [11–14]. Health facility delivery is an important aspect of maternal care because of the presence of skilled birth professionals [12], as well as an enabling environment where resources necessary for effective management of obstetric complications are available [15,16].

A WHO report showed a 50 percent reduction in Egypt’s maternal mortality when the number of births assisted by qualified professionals doubled [17]. This implies that maternal and neonatal mortality in Nigeria can be reduced by ensuring that women have better maternal healthcare services, especially health facility delivery [18,19]. Although home delivery y may be relatively cheaper for these women, evidence suggests that homedelivery can pose a serious health risk to both the mother and infant [20–22].

Other than financial constraints, other identified factors for poor utilization of maternal health services in SSA include maternal age, maternal and partner literacy level, poor knowledge about obstetric danger signs, cultural beliefs, poor referral practices, scarcity of trained health workers, and poor coordination among staff [18,19]. These barriers have been found in Nigeria [11,23,24].

Studies in the past have suggested age at first delivery as a determinant of health facility delivery in Nigeria [11,23]. For instance, a study conducted among women in a semi-urban settlement in Zaria, Northern Nigeria, found that 77% of women who had first pregnancies before age 18 delivered at home compared to their older women counterparts with 56% home deliveries [11]. This implies that younger women in Nigeria tend to deliver at home compared to older women [11,23].

Similarly, the 2013 NDHS conducted estimated that 23% of Nigerian women aged 15–19 had begun childbearing, of which 17 percent out of this had their first child, and 5% were pregnant with their first child [25]. The 2018 NDHS also showed that the median age at first birth among women age 25–49 was 20.4 years. This means that half of the women aged 25–49 gave birth for the first time before age 21 [6].

Therefore, there is a need to examine how age at first birth is associated with health facility delivery in Nigeria. Hence, this study aimed at examining the role age at first birth plays in health facility delivery among women of reproductive age in Nigeria. The result of this study would be useful to public health policymakers in building the right approaches to take in encouraging health facility delivery in Nigeria.

## Materials and methods

### Study design and data source

This study involved a cross-sectional analysis of secondary data from NDHS conducted in 2018. We utilized data from the birth records (NGIR7ADT) in the women’s file. According to Corsi and Neuman [26], the Demographic Health Survey (DHS) is a nationally representative survey usually carried out to estimate sociodemographic, health, and other health-related indicators. The survey collects information on several indicators, including the place of childbirth and age at first childbirth. The survey is conducted in over 85 LMICs globally [26]. The 2018 NDHS employed a two-stage stratified sampling technique to recruit the respondents for the survey. A detailed explanation of the sampling procedures can be found in Aliaga and Ruilin [27] study and the 2018 NDHS report [6]. The 2018 NDHS dataset consisted of 41,821 individual women aged 15–49. 40,666 occupied households were selected for the sample, and 40,427 were interviewed successfully, resulting in a response rate of 99% [6]. Out of this, 34,193 of the respondents with complete cases on the variables of interest in this study were included in the final analysis. We relied on strengthening the reporting of observational studies in Epidemiology in writing this manuscript [28].

### Variables

#### Outcome variable

The main outcome variable was the place of delivery. NDHS asks questions on place of delivery within five years preceding the survey, and the response options received were respondent’s home, other homes, government hospital, government health center, government health post, other public sectors, private hospital/clinic, other private sectors, and other health facilities. Those who responded respondent’s home and other homes were classified as “home delivery,” while the remaining responses were grouped as “health facility delivery”. This classification was informed by previous studies [29–32].

#### Key explanatory variable

The main explanatory variable was the age at first childbirth. This was assessed using the variable “age of respondent at first childbirth”. The response options ranged from 12 years to 48 years. The age at first childbirth was then created by recoding the responses into “below 20 years” and “20 years and above”. This categorization was informed by previous findings on age at first birth in Nigeria [6,25] and elsewhere [33].

#### Covariates

A total of thirteen covariates were studied. The covariates were selected based on their significant association with health facility delivery [29,30,34,35] and their availability in the DHS dataset. The covariates include maternal age, place of residence, parity, level of education, wealth quintile, current working status, marital status, religion, visit to the health facility in the past 12 months, permission to get medical help, distance to the health facility, region, and exposure to media. For this study, several of the covariates were recoded. The pre-existing coding for the place of residence, distance to the health facility, permission to get medical help, region, and visited health facility last 12 months were maintained and used in the final analysis. Maternal age was recoded into “15–24”, “25–34”, and “35+”. The level of education was re-classified into “no education”, “primary”, and “secondary or higher”. Wealth index was recoded as “poor”, “middle’, and “rich” by recoding poorer wealth quantile and poorest wealth quantile as “poor”, richer wealth quantile and richest wealth quantile as “Rich”, Middle wealth quantile still retains the categorisation as “Middle”. This categorization has been utilized elsewhere as well [36,37]. Also, marital status was recoded as “single”, “married”, and “others”. Parity was re-grouped into “1–3”, “4–6”, and “7+”. The women’s current working status was ground into “yes” and “no”. Religious affiliation of the women was recoded as “Christian”, “Islam” and traditional and other religious were recategorized as “others”. Exposure to media was created from three (3) variables (frequency of watching television, frequency of reading newspaper/magazine, and frequency of listening to the radio). All the three variables had the same response options (not at all less than once a week and at least once a week). The responses were recoded as “No = not at all” and “Yes” (less than once a week and at least once a week). Later, an index called the media exposure variable was created. In the current study, media exposure was defined as a woman with exposure in all three variables (watching television, reading newspaper/magazine, and listening to the radio).

### Data analysis

Data were analyzed using Stata version 16.0 (Stata Corporation, College Station, TX, USA). A descriptive analysis was first performed to determine the proportion of health facility delivery, with the result presented using percentages. A Pearson chi-square test analysis was later carried out to examine the relationship between health facility and age at first birth and the studied covariates. Also, bivariate and multivariable binary logistic regression analyses were performed to determine the association between health facility and age at first birth and selected covariates. The results were presented using crude odds ratio [38] and adjusted odds ratio (aOR) at 95% confidence intervals (CIs). The level of statistical significance was set at p<0.05 in the chi-square and regression analyses. A multicollinearity test was conducted using the Variance Inflation Factor (VIF), and we found no evidence of multicollinearity among the studied variables. During the analysis, the women’s sample weights (v005/1,000,000) were applied to obtain unbiased estimates. According to the DHS guidelines and the survey command (SVY) in Stata, it was used to adjust the data’s complex sampling structure in both the chi-square and regression analyses.

### Ethical approval

This was a secondary analysis of data, and therefore no ethical approval was required since the data is available in the public domain. Further information about the DHS data usage and ethical standards is available at http://goo.gl/ny8T6X. The authors of this manuscript did not collect the data, we sought permission from the MEASURE DHS website, access to the latest dataset of Nigeria was provided after our intent for the request was assessed and approved on the 20th of March 2021. The DHS surveys are ethically accepted by the ORC Macro Inc. Ethics Committee and the Ethics Boards of partner organizations in different countries, such as the Ministries of Health. The interviewed women gave either written or verbal consent during each of the surveys and followed Helsinki’s declaration of ethical principles.

## Results

### Distribution of key independent variable, covariate, and place of delivery

Table 1 presents results on the distribution of age at first childbirth, covariates, and place of delivery among women of reproductive ages in Nigeria. The prevalence of health facility deliveries was 41%. Age at first birth shows that 72.94% of home deliveries were among women who had their first birth below the age of 20. The chi-square (χ2) results showed a significant relationship (p<0.001) between age at first childbirth all selected co-variates, and place of delivery.

**Table 1:** Weighted distribution of key independent and covariate variables and place of delivery (n = 34,193).

Variables	Percentage (%)	Place of delivery (%)		p-value (χ^2^)
		Home	Health facility	
**Age at first birth**				<0.001
20 years and above	42.04	39.80	60.20	
Below 20 years	57.96	72.94	27.06	
**Age**				<0.001
15–24	23.82	66.25	33.75	
25–34	50.84	56.20	43.80	
35 +	25.34	57.82	42.18	
**Place of residence**				<0.001
Urban	38.52	36.34	63.66	
Rural	61.48	73.20	26.8	
**Parity**				<0.001
1–3	46.46	49.07	50.93	
4–6	34.29	61.19	38.81	
7+	19.25	79.09	20.91	
**Level of education**				<0.001
No education	46.38	85.98	14.02	
Primary	14.93	56.4	43.60	
Secondary and above	38.70	27.68	72.32	
**Wealth index**				<0.001
Poor	44.91	83.06	16.94	
Middle	20.60	58.08	41.92	
Rich	34.50	28.24	71.76	
**Currently working**				<0.001
No	32.41	71.32	28.68	
Yes	67.59	53.10	46.9	
**Marital status**				<0.001
Single	1.71	40.89	59.11	
Married	92.63	60.08	39.92	
Others	5.66	46.08	53.20	
**Religion**				<0.001
Christian	35.99	30.85	69.15	
Islam	63.48	74.83	25.17	
Others	0.53	75.30	24.70	
**Visited health facility last 12 months**				<0.001
No	46.68	65.42	34.58	
Yes	53.32	53.39	46.61	
**Region**				<0.001
North Central	13.51	50.13	49.87	
North East	18.17	74.50	25.50	
North West	36.73	84.42	15.58	
South East	10.02	17.18	82.82	
South-South	8.68	43.83	56.17	
South West	12.89	16.79	83.21	
**Permission to get medical help**				<0.001
Big problem	11.80	72.40	27.60	
Not a big problem	88.20	57.21	42.79	
**Distance to the health facility**				<0.001
Big problem	28.09	69.3	30.7	
Not a big problem	71.91	54.98	45.02	
**Exposure to media**				<0.001
No	38.87	81.18	18.82	
Yes	61.13	44.9	55.10	
**Nigeria = 34,193**	**100**	**59.00**	**41.00**	

Weighted NDHS, 2018.

### Association between age at first birth and health facility delivery in Nigeria

Table 2 shows the results of age at first birth and co-variates associated with health facility delivery in Nigeria. There is a significant relationship between the key independent variable (age at first birth) and health facility delivery among women of reproductive age in Nigeria. The adjusted odds ratio shows that women who had their first birth below age 20 [aOR = 0.82; 95% (CI = 0.74–0.90)] were less likely to give birth at health facilities compared to those who had their first birth at age 20 and above. Among the selected co-variates, age of respondents, place of residence, parity, level of education, wealth index, currently working, religion, region, visit to health facility, Permission to get Medical help, distance to health facility and exposure to media were significant with health facility delivery.

**Table 2:** Bivariate and multivariable logistics regression results on the predictors of health facility delivery (n = 34,193).

Variables	Unadjusted		Adjusted	
	cOR	95% CI	aOR	95% CI
**Age at first birth (RC = 20 year & above)**				
Below 20 years	0.24***	0.22–0.27	0.82***	0.74–0.90
**Age group (RC = 15–24)**				
25–34	1.53***	1.39–1.68	1.01	0.90–1.13
35 +	1.43***	1.28–1.60	1.21*	1.03–1.42
**Place of residence (RC = Urban)**				
Rural	0.21***	0.17–0.25	0.79**	0.68–0.92
**Parity (RC = 1–3 children)**				
4–6	0.61***	0.57–0.66	0.78***	0.70–0.87
7+	0.25***	0.23–0.28	0.65***	0.55–0.76
**Level of education (RC = No education)**				
Primary	4.74***	0.06–5.53	1.74***	1.53–1.97
Secondary and above	16.03***	13.90–18.47	2.82***	2.44–3.25
**Wealth status (RC = Poor)**				
Middle	3.54***	2.48–3.18	1.70***	1.50–1.93
Rich	12.46***	10.57–14.69	2.69***	2.32–3.12
**Currently working (RC = No)**				
Yes	2.20***	1.99–2.43	1.17**	1.06–1.30
**Marital status (RC = Single)**				
Married	0.46***	0.37–0.57	1.24	0.98–1.57
Others	0.79	0.61–1.01	1.05	0.79–1.39
**Religion (RC = Christian)**				
Islam	0.15***	0.13–0.18	0.58***	0.48–0.69
Others	0.15***	0.08–0.28	0.43**	0.26–0.70
**Region (**RC = North Central)				
North East	0.34***	0.27–0.44	0.65***	0.53–0.78
North West	0.19***	0.14–0.24	0.32***	0.27–0.39
South East	4.85***	3.53–6.66	1.69***	1.10–1.51
South South	1.29*	1.00–1.65	0.44***	0.43–0.64
South West	4.98***	3.85–6.45	2.14***	1.11–1.53
**Visited health facility in last 12 months (RC = No)**				
Yes	1.65***	1.50–1.81	1.46***	1.33–1.60
**Permission to get Medical help (RC = Big problem)**				
Not a big problem	1.96***	1.63–2.36	1.30***	1.12–1.51
**Distance to health facility (RC = Big problem)**				
Not a big problem	1.85***	1.62–2.11	1.30***	1.16–1.45
**Exposure to media (RC = No)**				
Yes	5.29***	4.71–5.95	1.44***	1.30–1.60

Weighted NDHS, 2018.

RC = Recode; cOR = unadjusted odds ratios; aOR = adjusted odds ratios; CI = confidence interval.

* p < 0.05

** p< 0.01

*** p < 0.001.

## Discussion

This study assessed the age at first birth and its association with health facility delivery among women of reproductive age in Nigeria. This is important to prioritize the healthcare needs of women of reproductive age, considering that place of delivery determines the quality of care received by women of reproductive age in Nigeria.

Our study showed a prevalence of 59% homedeliveries in Nigeria among women of reproductive-aged 15–49. This result is higher than a study conducted by Olusanya and Alakija [39] that reported 51.4% of non-utilization of facility-based maternity services in Lagos, Nigeria. In the same vein, the study result is higher compared to a study conducted in Tanzania that reported 35.5% [40] but lower than a study conducted in Ethiopia with 81.1% prevalence of homedelivery among the same characteristics of women [41].

We also found a significant relationship between the key independent variable (age at first birth) and health facility delivery among women of reproductive age in Nigeria. The adjusted odds ratio showed that women who had their first birth below age 20 were less likely to give birth at health facilities compared to those who had their first birth at age 20 and above. This could be due to the stigmatization that goes with unintended pregnancy outside marriage, and the women might prefer home delivery to avoid being stigmatized by the health attendants. Similar studies in sub-Saharan Africa showed that choosing a health facility as the place of delivery among women of reproductive age increased with age [42,43].

Among the selected co-variates, age of respondents, place of residence, parity, level of education, wealth index, currently working, religion, region, visit to the health facility, permission to get medical help, distance to health facility and exposure to media were significantly associated with health facility delivery.

Our study showed that respondents who were residing in the rural area of Nigeria were less likely to give birth at the health facility than those from the urban area. This is evidently showing the rural-urban inequality that exists in accessing health care facilities. Rural residents face numerous barriers to healthcare, which limits them from conveniently accessing and obtaining the appropriate care needed [20,44].

Women with number of children between 4 and 6 were less likely to give birth at the health facility alongside women with 7 children and above. Inadequate resources resulting from large family size and negative experiences from previous pregnancies have been identified as barriers to seeking maternal healthcare services by women with many children [45,46].

Religious beliefs and practices significantly determine a woman’s place of birth delivery. Women practicing Islam were less likely to give birth at the health facility compared to their Christian counterparts. This finding is based on religious grounds, whereby women who practice Islam prefer a female birth attendant for delivery, hence, avoid visiting health care facilities so as not to be attended to by a male healthcare worker [47–50].

In the same vein, the women who reside in the North East and South-South regions of Nigeria were less likely to give birth at the health facilities compared to those residing in the South West region. Despite the fact that the South-South region of Nigeria is rich in oil, yet the rate of facility delivery is low within the region [31,46]. This could be due to the disparities in socioeconomic status, distribution of healthcare services, and culture in Nigeria’s various regions [51]. Similarly, a study conducted in Kenya found a disparity in place of delivery by region as women residing in the Northern region were less likely to utilise health facilities for delivery [52].

We found that the odds of choosing to give birth at the health facility were higher among women age 35 and above. This is contrary to another study conducted in SSA, which showed that the odds of choosing a health facility as the place of delivery declined with age among reproductive age women [29]. Women who had secondary education and above were more likely to give birth at the health facility than women with primary education or no education. This shows that the level of education can influence a woman’s choice of a place to give birth. This finding is consistent with studies that reported that a woman’s educational status is significantly associated with the health facility delivery [53–56].

Our study also showed that rich women were more likely to give birth at Nigeria’s health facilities. This is in concordance with a study conducted in Ghana that wealthy women were more likely to utilize health facilities for birth delivery [57]. Similarly, women who are currently working were more likely to give birth at the health facility. The out-of-pocket payment system for healthcare services poses a huge barrier for unemployed women who do not have any means of livelihood to access the health facility to give birth, which also affects poor women [51].

The findings of this research showed that women residing closer to the health facility were more likely to give birth at the health facility than women who reside far from the health facility. The distance to health facilities has been recognized as a determining factor for the previously utilized healthcare facilities [58]. Also, women who were exposed to mass media were more likely to give birth at the health facilities than their counterparts who were not exposed to mass media. This shows that the mass media plays a significant role in positively influencing the better practice of women’s reproductive health behavior [29,59]. This aligns with a spatial analysis study conducted in Nigeria in 2014 [46] and a recent study conducted in Ethiopia [60].

### Strengths and limitations

This study’s major strength is using the current nationally representative survey, which makes the study’s findings generalizable to women of reproductive age in Nigeria. Despite this strength, there are some limitations inherent in this study that must be acknowledged. Firstly, because the age at first childbirth and place of delivery were both self-reported, this study is subject to recall bias. Thus, respondents may over-or under-report their age at first childbirth or the place of delivery which could affect the findings’ interpretations. Secondly, the study dataset was cross-sectional, so it was only possible to conclude an association between age at first childbirth and health facility delivery and not causality. Thirdly, Nigeria is diverse, and the geopolitical zones have socio-cultural differences dating back to precolonial times; hence, the finding on age at first childbirth (and other covariates) may not hold across all the six zones. Hence, future studies may consider addressing this limitation.

## Conclusion and recommendation

Our findings call for the need to implement health education programmes among women of reproductive age in Nigeria to increase awareness on the importance of giving birth at the health facilities to avoid the risks of maternal complications associated with home delivery. These health education programmes should be targeted at women of reproductive age younger than 20 years, those with no education, those currently not working, the poor, those currently residing in the Northern part of the country, those practicing Islam and those not exposed to mass media. Since one of the factors contributing to homedelivery was wealth index, and those who are in the poor wealth index group were less likely to utilize healthcare facilities for delivery, then Nigeria government and non-governmental organization should make healthcare payment more affordable for every woman of reproductive age by establishing a separate health payment scheme that will be targeted to women within poor wealth index.
